# Tris[4-bromo-2-(methyl­imino­meth­yl)phenolato-κ^2^ *N*,*O*]cobalt(III)

**DOI:** 10.1107/S1600536813027591

**Published:** 2013-10-16

**Authors:** Qiu-Ping Huang, Chun-Lian Zhang, Ru-Xia Zhao, Li Yang, Xi-Fu Jiang

**Affiliations:** a College of Chemistry and Bioengineering, Guilin University of Technology, Guilin 541004, People’s Republic of China

## Abstract

In the title compound, [Co(C_8_H_7_BrNO)_3_], the Co^III^ ion is coordinated in a slightly distorted octa­hedral environment by three N atoms and three O atoms from three bidentate 4-bromo-2-(methyl­imino­meth­yl)phenolate ligands. The dihedral angles between the benzene rings are 82.6 (2), 57.1 (2) and 62.9 (2)°. In the crystal, mol­ecules are linked by pairs of weak C—H⋯Br hydrogen bonds, forming inversion dimers.

## Related literature

For applications of Schiff base complexes, see: Pradeep & Das (2013 ▶); Shankara *et al.* (2013 ▶); Feng *et al.* (2007 ▶); Yang *et al.* (2007 ▶); Raptopoulou *et al.* (2006 ▶); Zhang & Feng (2010 ▶); Qin *et al.* (2009 ▶). For related structures, see: Park *et al.* (2008 ▶); Huang *et al.* (2011 ▶, 2012 ▶).
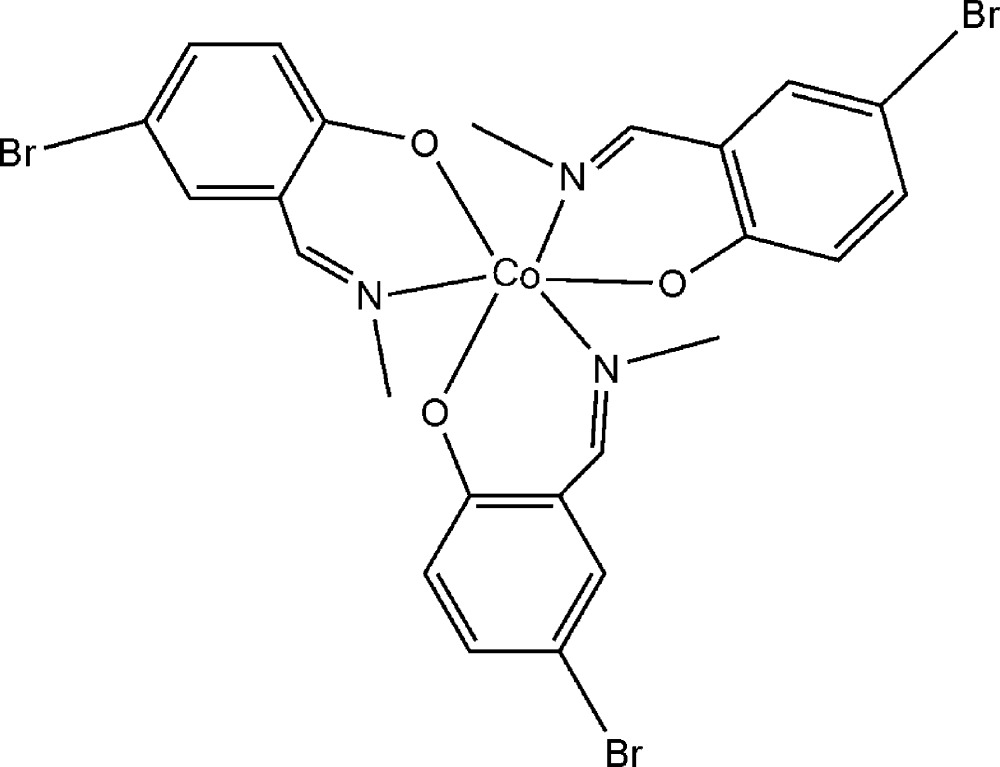


## Experimental

### 

#### Crystal data

[Co(C_8_H_7_BrNO)_3_]
*M* *_r_* = 698.10
Orthorhombic, 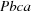

*a* = 17.1086 (6) Å
*b* = 15.0188 (4) Å
*c* = 19.9578 (7) Å
*V* = 5128.2 (3) Å^3^
*Z* = 8
Mo *K*α radiation
μ = 5.38 mm^−1^
*T* = 293 K
0.18 × 0.16 × 0.14 mm

#### Data collection

Bruker SMART CCD diffractometer
Absorption correction: multi-scan (*SADABS*; Bruker, 2004 ▶) *T*_min_ = 0.282, *T*_max_ = 1.000
15312 measured reflections
4558 independent reflections
3104 reflections with *I* > 2σ(*I*)
*R*_int_ = 0.033

#### Refinement

*R*[*F* ^2^ > 2σ(*F* ^2^)] = 0.041
*wR*(*F* ^2^) = 0.089
*S* = 1.01
4558 reflections
310 parameters
H-atom parameters constrained
Δρ_max_ = 0.82 e Å^−3^
Δρ_min_ = −0.75 e Å^−3^

### 

Data collection: *SMART* (Bruker 2004 ▶); cell refinement: *SAINT* (Bruker, 2004 ▶); data reduction: *SAINT*; program(s) used to solve structure: *SHELXS97* (Sheldrick, 2008 ▶); program(s) used to refine structure: *SHELXL97* (Sheldrick, 2008 ▶); molecular graphics: *SHELXTL* (Sheldrick, 2008 ▶) and *PLATON* (Spek, 2009 ▶); software used to prepare material for publication: *SHELXL97*.

## Supplementary Material

Crystal structure: contains datablock(s) global, I. DOI: 10.1107/S1600536813027591/lh5657sup1.cif

Structure factors: contains datablock(s) I. DOI: 10.1107/S1600536813027591/lh5657Isup2.hkl

Supplementary material file. DOI: 10.1107/S1600536813027591/lh5657Isup3.cdx

Additional supplementary materials:  crystallographic information; 3D view; checkCIF report

## Figures and Tables

**Table 1:** Hydrogen-bond geometry (Å, °)

*D*—H⋯*A*	*D*—H	H⋯*A*	*D*⋯*A*	*D*—H⋯*A*
C19—H19⋯Br1^i^	0.93	2.90	3.657 (4)	140

Symmetry code: (i) 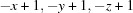.

**Fig. 1.**
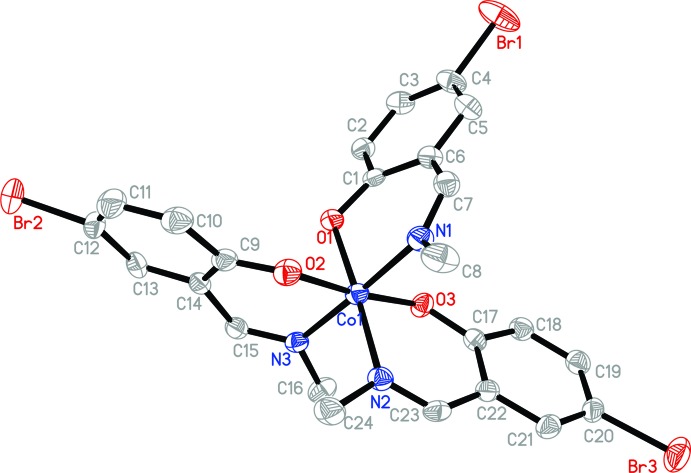
Molecular structure of the title compound with displacement ellipsoids drawn at the 30% probability level

**Fig. 2.**
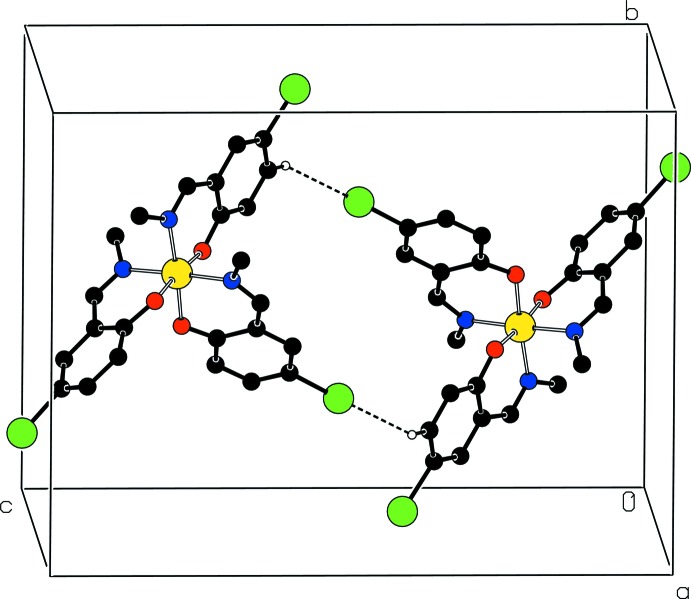
An inversion dimer of (I) with weak hydrogen bonds shown as dashed lines. Only H atoms involved in weak hydrogen bonds are shown.

## supplementary crystallographic information

### 1. Comment

Schiff base complexes play an important role in antibacterial and catalytic performance, and have attracted widespread interest by researchers (Pradeep & Das, 2013; Shankara *et al.*, 2013). In addition, Schiff base complexes are of great significance in the biological and medical field (Feng *et al.*, 2007; Yang *et al.*, 2007; Raptopoulou *et al.*, 2006; Zhang *et al.*, 2010; Qin *et al.*, 2009). The crystal structures of some related Co(III) complexes already appear in the literature (Park, *et al.*, 2008; Huang, *et al.*, 2011,2012). In the title complex, the Co^III^ ion is in a slightly distorted octahedral geometry, coordinated by three N atoms and three O atoms from three bidentate 4-bromo-2-(methyliminomethyl)phenolate ligands (Fig. 1). The dihedral angles between the benzene rings are 82.6 (2)° [C1-C6/C9-C14], 57.1 (2)° [C1-C6/C17-C22] and 62.9 (2)° [C9-C14/C17-C22]. The Co ion is in the 3+ oxidation state, as evidenced by bond valence summation calculations, charge balance considerations, and the presence of typical bond lengths for a Co^III^ ion (Park, *et al.* 2008; Huang, *et al.* 2012). In the crystal, molecules are linked by a pair of weak C—H···Br hydrogen bonds forming inversion dimers (Fig. 2).

### 2. Experimental

The title compound was prepared from a mixture of 5-bromo-2-hydroxy-benzaldehyde (0.181 g, 1.0 mmol), methylamine (0.031 g, 1.0 mmol), sodium hydroxide (0.040 g, 1 mmol), cobalt nitrate hexahydrate (0.145 g, 0.5 mmol) and methanol (8 ml), sealed in a 20 ml Teflon-lined stainless steel bomb, and kept at 373K for 3 days under autogenous pressure. After the reaction was slowly cooled to room temperature, brown block-like crystals were obtained (yield: 63% based on cobalt). *Anal. Calc*. for C_24_H_21_N_3_O_3_Br_3_Co (%): C, 41.15; H, 3.01; N, 6.02. Found (%): C, 41.17; H, 3.12; N, 6.00.

### 3. Refinement

H atoms were positioned geometrically and refined with a riding model, with C–H distances of 0.93–0.96 Å, with *U*_iso_(H) = 1.2 *U*_eq_(C) or 1.5 *U*_eq_(C) for methyl H atoms.

### Crystal data

**Table:**

[Co(C_8_H_7_BrNO)_3_]	*F*(000) = 2736
*M**_r_* = 698.10	*D*_x_ = 1.808 Mg m^−^^3^
Orthorhombic, *P**b**c**a*	Mo *K*α radiation, λ = 0.71073 Å
Hall symbol: -P 2ac 2ab	Cell parameters from 4287 reflections
*a* = 17.1086 (6) Å	θ = 2.9–29.1°
*b* = 15.0188 (4) Å	µ = 5.38 mm^−^^1^
*c* = 19.9578 (7) Å	*T* = 293 K
*V* = 5128.2 (3) Å^3^	Block, brown
*Z* = 8	0.18 × 0.16 × 0.14 mm

### Data collection

**Table:**

Bruker SMART CCD diffractometer	4558 independent reflections
Radiation source: fine-focus sealed tube	3104 reflections with *I* > 2σ(*I*)
Graphite monochromator	*R*_int_ = 0.033
Detector resolution: no pixels mm^-1^	θ_max_ = 25.1°, θ_min_ = 2.9°
ω scans	*h* = −19→20
Absorption correction: multi-scan (*SADABS*; Bruker, 2004)	*k* = −17→17
*T*_min_ = 0.282, *T*_max_ = 1.000	*l* = −22→23
15312 measured reflections	

### Refinement

**Table:**

Refinement on *F*^2^	Primary atom site location: structure-invariant direct methods
Least-squares matrix: full	Secondary atom site location: difference Fourier map
*R*[*F*^2^ > 2σ(*F*^2^)] = 0.041	Hydrogen site location: inferred from neighbouring sites
*wR*(*F*^2^) = 0.089	H-atom parameters constrained
*S* = 1.01	*w* = 1/[σ^2^(*F*_o_^2^) + (0.030*P*)^2^ + 7.4066*P*] where *P* = (*F*_o_^2^ + 2*F*_c_^2^)/3
4558 reflections	(Δ/σ)_max_ < 0.001
310 parameters	Δρ_max_ = 0.82 e Å^−^^3^
0 restraints	Δρ_min_ = −0.75 e Å^−^^3^

### Special details

**Table:**

Geometry. All e.s.d.'s (except the e.s.d. in the dihedral angle between two l.s. planes) are estimated using the full covariance matrix. The cell e.s.d.'s are taken into account individually in the estimation of e.s.d.'s in distances, angles and torsion angles; correlations between e.s.d.'s in cell parameters are only used when they are defined by crystal symmetry. An approximate (isotropic) treatment of cell e.s.d.'s is used for estimating e.s.d.'s involving l.s. planes.
Refinement. Refinement of *F*^2^ against ALL reflections. The weighted *R*-factor *wR* and goodness of fit *S* are based on *F*^2^, conventional *R*-factors *R* are based on *F*, with *F* set to zero for negative *F*^2^. The threshold expression of *F*^2^ > σ(*F*^2^) is used only for calculating *R*-factors(gt) *etc*. and is not relevant to the choice of reflections for refinement. *R*-factors based on *F*^2^ are statistically about twice as large as those based on *F*, and *R*- factors based on ALL data will be even larger.

### Fractional atomic coordinates and isotropic or equivalent isotropic displacement parameters (Å^2^)

**Table:**

	*x*	*y*	*z*	*U*_iso_*/*U*_eq_	
Br1	0.42428 (4)	0.69768 (5)	0.48113 (3)	0.0903 (2)	
Br2	0.19400 (4)	0.72733 (4)	−0.03750 (3)	0.0880 (2)	
Br3	0.45860 (4)	0.03597 (4)	0.40953 (3)	0.0764 (2)	
C1	0.3526 (2)	0.5538 (2)	0.2818 (2)	0.0346 (10)	
C2	0.4091 (3)	0.6212 (3)	0.2826 (2)	0.0422 (11)	
H2	0.4324	0.6387	0.2426	0.051*	
C3	0.4310 (3)	0.6622 (3)	0.3414 (3)	0.0492 (12)	
H3	0.4684	0.7071	0.3409	0.059*	
C4	0.3973 (3)	0.6367 (3)	0.4009 (2)	0.0529 (13)	
C5	0.3430 (3)	0.5699 (3)	0.4029 (2)	0.0503 (12)	
H5	0.3207	0.5534	0.4435	0.060*	
C6	0.3210 (3)	0.5265 (3)	0.3439 (2)	0.0409 (11)	
C7	0.2603 (3)	0.4607 (3)	0.3472 (2)	0.0457 (12)	
H7	0.2331	0.4560	0.3874	0.055*	
C8	0.1706 (3)	0.3516 (3)	0.3114 (3)	0.0638 (15)	
H8A	0.1482	0.3657	0.3542	0.096*	
H8B	0.1856	0.2900	0.3106	0.096*	
H8C	0.1327	0.3625	0.2768	0.096*	
C9	0.2016 (3)	0.5015 (3)	0.1256 (2)	0.0432 (11)	
C10	0.1327 (3)	0.5518 (3)	0.1133 (3)	0.0553 (13)	
H10	0.0884	0.5414	0.1392	0.066*	
C11	0.1304 (3)	0.6153 (3)	0.0641 (3)	0.0651 (16)	
H11	0.0847	0.6472	0.0566	0.078*	
C12	0.1959 (3)	0.6321 (3)	0.0255 (3)	0.0562 (14)	
C13	0.2619 (3)	0.5815 (3)	0.0326 (2)	0.0496 (12)	
H13	0.3047	0.5912	0.0049	0.060*	
C14	0.2648 (3)	0.5150 (3)	0.0818 (2)	0.0396 (11)	
C15	0.3322 (3)	0.4577 (3)	0.0834 (2)	0.0389 (10)	
H15	0.3662	0.4608	0.0471	0.047*	
C16	0.4181 (3)	0.3453 (3)	0.1208 (3)	0.0512 (13)	
H16A	0.4374	0.3515	0.0758	0.077*	
H16B	0.4036	0.2844	0.1286	0.077*	
H16C	0.4583	0.3623	0.1519	0.077*	
C17	0.4012 (2)	0.2962 (3)	0.2891 (2)	0.0375 (10)	
C18	0.4649 (3)	0.2921 (3)	0.3340 (2)	0.0446 (11)	
H18	0.4960	0.3422	0.3406	0.054*	
C19	0.4814 (3)	0.2151 (3)	0.3681 (2)	0.0480 (12)	
H19	0.5230	0.2135	0.3980	0.058*	
C20	0.4367 (3)	0.1399 (3)	0.3582 (2)	0.0470 (12)	
C21	0.3765 (3)	0.1399 (3)	0.3132 (2)	0.0456 (12)	
H21	0.3481	0.0880	0.3058	0.055*	
C22	0.3573 (3)	0.2179 (3)	0.2782 (2)	0.0380 (10)	
C23	0.2951 (3)	0.2142 (3)	0.2299 (2)	0.0403 (11)	
H23	0.2753	0.1581	0.2200	0.048*	
C24	0.2037 (3)	0.2610 (3)	0.1494 (3)	0.0543 (13)	
H24A	0.1961	0.1978	0.1464	0.081*	
H24B	0.2196	0.2837	0.1066	0.081*	
H24C	0.1556	0.2889	0.1627	0.081*	
Co1	0.29471 (3)	0.40287 (3)	0.21453 (3)	0.03520 (16)	
N1	0.2399 (2)	0.4078 (2)	0.29993 (18)	0.0424 (9)	
N2	0.2642 (2)	0.2802 (2)	0.19908 (18)	0.0389 (9)	
N3	0.34932 (18)	0.4031 (2)	0.13001 (17)	0.0351 (8)	
O1	0.32936 (17)	0.52222 (16)	0.22408 (14)	0.0401 (7)	
O2	0.20115 (16)	0.44434 (19)	0.17475 (16)	0.0457 (8)	
O3	0.38751 (16)	0.37176 (17)	0.25932 (14)	0.0409 (7)	

### Atomic displacement parameters (Å^2^)

**Table:**

	*U*^11^	*U*^22^	*U*^33^	*U*^12^	*U*^13^	*U*^23^
Br1	0.0634 (4)	0.1341 (6)	0.0734 (4)	−0.0235 (4)	0.0026 (4)	−0.0567 (4)
Br2	0.1194 (6)	0.0797 (4)	0.0650 (4)	0.0317 (4)	−0.0267 (4)	0.0175 (3)
Br3	0.0879 (4)	0.0639 (3)	0.0773 (4)	0.0232 (3)	0.0011 (4)	0.0260 (3)
C1	0.038 (2)	0.024 (2)	0.042 (3)	0.0048 (19)	−0.002 (2)	−0.0038 (19)
C2	0.043 (3)	0.038 (2)	0.045 (3)	0.001 (2)	0.004 (2)	0.000 (2)
C3	0.040 (3)	0.047 (3)	0.061 (3)	−0.006 (2)	0.000 (3)	−0.011 (2)
C4	0.044 (3)	0.064 (3)	0.051 (3)	−0.003 (3)	−0.006 (3)	−0.023 (3)
C5	0.048 (3)	0.063 (3)	0.040 (3)	−0.002 (3)	0.003 (3)	−0.010 (2)
C6	0.041 (3)	0.039 (2)	0.043 (3)	−0.002 (2)	0.004 (2)	−0.004 (2)
C7	0.049 (3)	0.045 (3)	0.043 (3)	−0.002 (2)	0.007 (3)	0.000 (2)
C8	0.067 (3)	0.057 (3)	0.068 (4)	−0.024 (3)	0.022 (3)	−0.014 (3)
C9	0.043 (3)	0.039 (2)	0.048 (3)	0.008 (2)	−0.013 (3)	−0.011 (2)
C10	0.045 (3)	0.055 (3)	0.066 (4)	0.008 (3)	−0.004 (3)	−0.013 (3)
C11	0.060 (4)	0.060 (3)	0.075 (4)	0.019 (3)	−0.025 (3)	−0.009 (3)
C12	0.070 (4)	0.048 (3)	0.051 (3)	0.014 (3)	−0.027 (3)	−0.002 (2)
C13	0.058 (3)	0.058 (3)	0.032 (3)	0.009 (3)	−0.013 (3)	−0.003 (2)
C14	0.044 (3)	0.042 (3)	0.033 (2)	0.007 (2)	−0.007 (2)	−0.007 (2)
C15	0.041 (3)	0.041 (2)	0.035 (3)	0.002 (2)	0.000 (2)	−0.005 (2)
C16	0.041 (3)	0.044 (3)	0.069 (3)	0.009 (2)	0.011 (3)	0.003 (2)
C17	0.036 (2)	0.043 (3)	0.034 (2)	0.000 (2)	0.002 (2)	−0.003 (2)
C18	0.046 (3)	0.044 (3)	0.043 (3)	−0.003 (2)	−0.003 (3)	−0.004 (2)
C19	0.047 (3)	0.055 (3)	0.042 (3)	0.010 (2)	−0.004 (3)	−0.005 (2)
C20	0.058 (3)	0.044 (3)	0.038 (3)	0.021 (3)	0.012 (3)	0.007 (2)
C21	0.051 (3)	0.038 (2)	0.047 (3)	0.005 (2)	0.012 (3)	0.002 (2)
C22	0.041 (2)	0.035 (2)	0.038 (3)	0.000 (2)	0.003 (2)	−0.002 (2)
C23	0.043 (3)	0.030 (2)	0.048 (3)	−0.007 (2)	0.006 (3)	−0.008 (2)
C24	0.051 (3)	0.052 (3)	0.060 (3)	−0.004 (3)	−0.016 (3)	−0.012 (2)
Co1	0.0362 (3)	0.0308 (3)	0.0386 (3)	−0.0002 (3)	−0.0013 (3)	−0.0029 (2)
N1	0.046 (2)	0.036 (2)	0.044 (2)	−0.0077 (18)	0.003 (2)	−0.0010 (18)
N2	0.036 (2)	0.040 (2)	0.040 (2)	−0.0007 (17)	−0.0041 (19)	−0.0048 (17)
N3	0.0316 (19)	0.0326 (18)	0.041 (2)	0.0023 (16)	−0.0029 (18)	−0.0053 (17)
O1	0.0511 (18)	0.0315 (15)	0.0379 (18)	−0.0013 (14)	−0.0001 (16)	−0.0016 (13)
O2	0.0368 (17)	0.0480 (17)	0.052 (2)	0.0055 (15)	0.0010 (17)	−0.0023 (16)
O3	0.0433 (17)	0.0314 (15)	0.0480 (18)	−0.0062 (14)	−0.0108 (16)	0.0041 (14)

### Geometric parameters (Å, º)

**Table:**

Br1—C4	1.902 (4)	C14—C15	1.438 (6)
Br2—C12	1.904 (5)	C15—N3	1.275 (5)
Br3—C20	1.904 (4)	C15—H15	0.9300
C1—O1	1.308 (5)	C16—N3	1.475 (5)
C1—C2	1.400 (6)	C16—H16A	0.9600
C1—C6	1.413 (6)	C16—H16B	0.9600
C2—C3	1.377 (6)	C16—H16C	0.9600
C2—H2	0.9300	C17—O3	1.302 (5)
C3—C4	1.374 (6)	C17—C22	1.412 (6)
C3—H3	0.9300	C17—C18	1.413 (6)
C4—C5	1.368 (6)	C18—C19	1.371 (6)
C5—C6	1.396 (6)	C18—H18	0.9300
C5—H5	0.9300	C19—C20	1.378 (6)
C6—C7	1.436 (6)	C19—H19	0.9300
C7—N1	1.281 (5)	C20—C21	1.367 (6)
C7—H7	0.9300	C21—C22	1.404 (6)
C8—N1	1.474 (5)	C21—H21	0.9300
C8—H8A	0.9600	C22—C23	1.437 (6)
C8—H8B	0.9600	C23—N2	1.281 (5)
C8—H8C	0.9600	C23—H23	0.9300
C9—O2	1.304 (5)	C24—N2	1.462 (5)
C9—C14	1.404 (6)	C24—H24A	0.9600
C9—C10	1.421 (6)	C24—H24B	0.9600
C10—C11	1.370 (7)	C24—H24C	0.9600
C10—H10	0.9300	Co1—O3	1.881 (3)
C11—C12	1.383 (7)	Co1—O2	1.892 (3)
C11—H11	0.9300	Co1—O1	1.898 (3)
C12—C13	1.369 (6)	Co1—N3	1.928 (3)
C13—C14	1.402 (6)	Co1—N2	1.939 (3)
C13—H13	0.9300	Co1—N1	1.946 (4)
			
O1—C1—C2	118.8 (4)	O3—C17—C22	124.0 (4)
O1—C1—C6	123.5 (4)	O3—C17—C18	117.8 (4)
C2—C1—C6	117.6 (4)	C22—C17—C18	118.2 (4)
C3—C2—C1	121.3 (4)	C19—C18—C17	120.6 (4)
C3—C2—H2	119.3	C19—C18—H18	119.7
C1—C2—H2	119.3	C17—C18—H18	119.7
C4—C3—C2	119.9 (4)	C18—C19—C20	120.5 (4)
C4—C3—H3	120.1	C18—C19—H19	119.8
C2—C3—H3	120.1	C20—C19—H19	119.8
C5—C4—C3	121.0 (4)	C21—C20—C19	120.8 (4)
C5—C4—Br1	119.6 (4)	C21—C20—Br3	120.1 (4)
C3—C4—Br1	119.4 (4)	C19—C20—Br3	119.1 (4)
C4—C5—C6	120.0 (4)	C20—C21—C22	120.3 (4)
C4—C5—H5	120.0	C20—C21—H21	119.9
C6—C5—H5	120.0	C22—C21—H21	119.9
C5—C6—C1	120.1 (4)	C21—C22—C17	119.6 (4)
C5—C6—C7	118.5 (4)	C21—C22—C23	118.3 (4)
C1—C6—C7	121.1 (4)	C17—C22—C23	122.1 (4)
N1—C7—C6	126.2 (4)	N2—C23—C22	126.7 (4)
N1—C7—H7	116.9	N2—C23—H23	116.7
C6—C7—H7	116.9	C22—C23—H23	116.7
N1—C8—H8A	109.5	N2—C24—H24A	109.5
N1—C8—H8B	109.5	N2—C24—H24B	109.5
H8A—C8—H8B	109.5	H24A—C24—H24B	109.5
N1—C8—H8C	109.5	N2—C24—H24C	109.5
H8A—C8—H8C	109.5	H24A—C24—H24C	109.5
H8B—C8—H8C	109.5	H24B—C24—H24C	109.5
O2—C9—C14	124.6 (4)	O3—Co1—O2	174.38 (12)
O2—C9—C10	118.3 (4)	O3—Co1—O1	85.60 (12)
C14—C9—C10	117.1 (4)	O2—Co1—O1	89.74 (12)
C11—C10—C9	121.1 (5)	O3—Co1—N3	90.42 (13)
C11—C10—H10	119.4	O2—Co1—N3	92.41 (14)
C9—C10—H10	119.4	O1—Co1—N3	86.25 (13)
C10—C11—C12	120.3 (5)	O3—Co1—N2	93.85 (13)
C10—C11—H11	119.9	O2—Co1—N2	91.03 (14)
C12—C11—H11	119.9	O1—Co1—N2	175.83 (14)
C13—C12—C11	120.6 (5)	N3—Co1—N2	89.62 (14)
C13—C12—Br2	120.0 (4)	O3—Co1—N1	89.98 (14)
C11—C12—Br2	119.4 (4)	O2—Co1—N1	87.00 (14)
C12—C13—C14	119.9 (5)	O1—Co1—N1	91.51 (13)
C12—C13—H13	120.1	N3—Co1—N1	177.69 (14)
C14—C13—H13	120.1	N2—Co1—N1	92.63 (14)
C13—C14—C9	120.7 (4)	C7—N1—C8	117.3 (4)
C13—C14—C15	118.0 (4)	C7—N1—Co1	122.5 (3)
C9—C14—C15	121.2 (4)	C8—N1—Co1	120.1 (3)
N3—C15—C14	125.8 (4)	C23—N2—C24	117.7 (4)
N3—C15—H15	117.1	C23—N2—Co1	123.2 (3)
C14—C15—H15	117.1	C24—N2—Co1	119.1 (3)
N3—C16—H16A	109.5	C15—N3—C16	118.1 (4)
N3—C16—H16B	109.5	C15—N3—Co1	121.9 (3)
H16A—C16—H16B	109.5	C16—N3—Co1	119.7 (3)
N3—C16—H16C	109.5	C1—O1—Co1	121.8 (2)
H16A—C16—H16C	109.5	C9—O2—Co1	121.8 (3)
H16B—C16—H16C	109.5	C17—O3—Co1	125.8 (3)

### Hydrogen-bond geometry (Å, º)

**Table:**

*D*—H···*A*	*D*—H	H···*A*	*D*···*A*	*D*—H···*A*
C19—H19···Br1^i^	0.93	2.90	3.657 (4)	140

Symmetry code: (i) −*x*+1, −*y*+1, −*z*+1.
